# Microbial Resource Regimes Shape Early Survival, Growth, and Reproductive Morphology in a Phally Polymorphic Terrestrial Gastropod

**DOI:** 10.1007/s00248-026-02814-2

**Published:** 2026-06-19

**Authors:** Zofia Książkiewicz, Karolina Górzyńska, Ewa Kosicka, Łukasz Wejnerowski, Michał Rybak

**Affiliations:** 1 Department of General Zoology, Faculty of Biology, Adam Mickiewicz University, Uniwersytetu Poznańskiego 6, Poznań, 61–614 Poland; 2 Department of Systematic and Environmental Botany, Faculty of Biology, Adam Mickiewicz University, Uniwersytetu Poznańskiego 6, 61-614 Poznań, Poland; 3 Department of Bioenergetics, Faculty of Biology, Adam Mickiewicz University in Poznań, Uniwersytetu Poznańskiego 6, Poznań, 61-614 Poland; 4 Department of Hydrobiology, Faculty of Biology, Adam Mickiewicz University, Uniwersytetu Poznańskiego 6, Poznań, 61-614 Poland; 5 Department of Water Protection, Faculty of Biology, Adam Mickiewicz University, Poznań, Uniwersytetu Poznańskiego 6, Poznań, 61–614 Poland

**Keywords:** Early-life survival, Gastropods, Microbially mediated nutrition, Developmental plasticity, Reproductive morphology, Wetland ecosystems

## Abstract

Microorganisms are fundamental components of terrestrial ecosystems, acting not only as symbionts or pathogens but also as key nutritional resources for invertebrates. Despite their ubiquity, their role in shaping early-life survival and phenotypic variation in terrestrial animals remains poorly understood. Here, we investigated how microbial resource regimes act as early-life ecological filters influencing survival, growth, and reproductive morphology in the phally polymorphic wetland microsnail *Vertigo antivertigo*. Using eggs and early juveniles, life stages characterized by high natural mortality, we conducted a series of laboratory experiments manipulating access to the species’ fecal- and litter-associated bacteria, soil and litter fungi, and the cyanobacterium *Limnothrix* sp. Survival emerged as the most consistent response variable across treatments. Fungal supplementation combined with plant litter supported growth and survival to sexual maturity, whereas bacterial resources alone did not compensate for the absence of intact litter-associated microorganisms. Exposure to *Limnothrix* sp. resulted in severe growth suppression and complete juvenile mortality, irrespective of bacterial supplementation. Disrupted microbial conditions were also associated with shifts in reproductive morphology, including reduced or absent male copulatory organs. Our findings demonstrate that microbial resource regimes can function as strong early-life ecological filters, shaping survival, growth and reproductive morphology.

**Supplementary Information:**

The online version contains supplementary material available at https://doi.org/10.1007/s00248-026-02814-2.

## Introduction

Microorganisms are integral components of ecological systems, shaping the physiology, behaviour, and evolutionary trajectories of animals through diverse interactions [1, 2]. Beyond their roles as symbionts or pathogens, microbes form microbial resource regimes, defined here as assemblages of bacteria, fungi, and other microorganisms associated with organic matter. These assemblages function both as direct food resources and as key agents in the fragmentation and conditioning of detrital organic matter, thereby facilitating microbial colonization and nutrient mineralization [3]. Consequently, these regimes influence the availability and quality of food for invertebrates, which constitute a substantial component of detrital food webs [4]. Within these regimes, microorganisms colonizing organic matter can further enhance its nutritional quality by transforming otherwise low-quality substrates into more accessible food resources, a process often referred to as “trophic upgrading” [4].

Microbial resource regimes are likely to exert particularly strong effects during early life stages, which are often characterized by high mortality and strong selective filtering [5]. Processes operating at these stages determine which individuals reach maturity and can bias the distribution of phenotypes within populations. While previous work has primarily emphasized abiotic drivers acting as such filters, e.g. temperature or moisture in terrestrial invertebrates [6], the role of microbial resource regimes in shaping early-life survival, growth and development remains poorly understood.

The microbial resource-mediated effects may be especially important in some simultaneous hermaphrodites, which rely on limited resources while allocating limited energy between male and female reproductive functions. Such condition-dependent allocation is particularly evident in phally polymorphic species [7, 8], where individuals within a population vary in the development of male copulatory organs and, consequently, in their capacity to donate sperm. Three phally morphs are typically distinguished, all of which retain the ability to self-fertilize but differ in their functional roles during copulation with conspecifics [9]. Euphallic individuals possess a fully developed penis and can perform both male and female roles during mating; hemiphallic individuals have a vestigial penis and therefore function primarily as females; and aphallic individuals completely lack a penis and function exclusively as females during copulation [7, 9, 10]. Individuals differentiate into a specific phally morph during ontogeny, with both genetic background and environmental conditions contributing to phally morph expression. These morphs are discrete and, once established, are irreversible, with no subsequent resorption or development of male copulatory organs [7, 9, 11]. Such variation in reproductive morphology provides a useful framework for testing how environmental factors, including microbial resource regimes, influence developmental and reproductive outcomes. Theory predicts that shifts in resource availability can alter sex allocation and reproductive trait expression in hermaphrodites, allowing reproduction to remain viable under variable environmental conditions [12, 13]. Variation in reproductive morphology may, in turn, influence the genetic variability of offspring and population persistence [14].

Using the wetland microsnail *Vertigo antivertigo* (Draparnaud, 1801), a species exhibiting phally polymorphism, we experimentally manipulated access to microorganisms naturally occurring in its habitat, including the species’ fecal- and litter-associated bacteria, fungi, and the cyanobacterium *Limnothrix sp.* Focusing on eggs and juveniles, we tested how microbial resource regimes affect (1) early-life survival and shell growth, and (2) the expression and frequency of phally morphs. By experimentally linking microbial resource regimes to survival rates and reproductive morphology, this study examines how early developmental conditions influence life-history and reproductive variation in a hermaphroditic invertebrate.

We hypothesized that access to intact microbial resource regimes associated with natural litter would enhance survival and growth, whereas simplified or disrupted microbial regimes would lead to their reduction. Based on previous study on other *Vertigo* species [15], we further expected that fungal supplementation would positively affect development, while exposure to cyanobacteria, previously reported as toxic to vertebrates [16–19], would negatively affect survival and growth. In wetland habitats, cyanobacteria may occur in water periodically inundating the litter layer, where eggs are laid and juveniles develop [20], making early-life exposure to cyanobacteria ecologically plausible. Given the condition-dependent nature of sex allocation in hermaphrodites, we also predicted that variation in microbial resource regimes would be associated with shifts in reproductive morphology.

## Materials and Methods

### Study Species

**Fig. 1:**
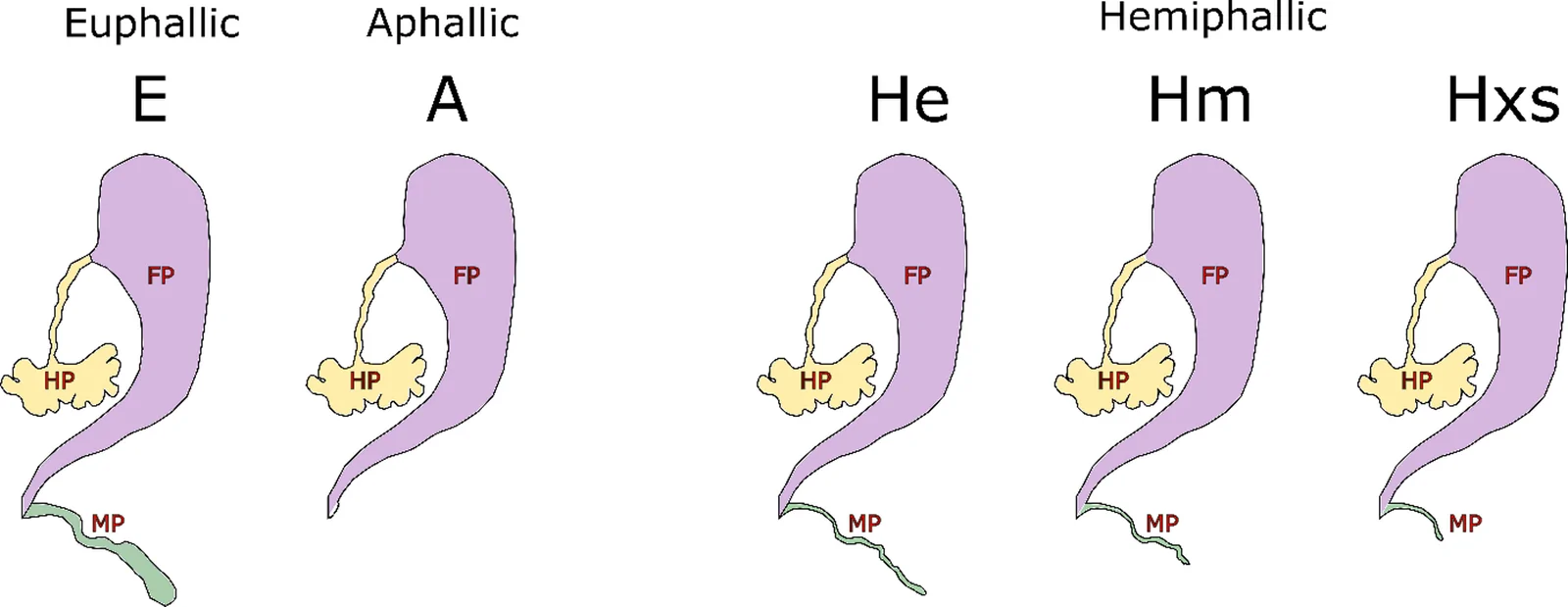
A schematic diagram of the reproductive tracts. HP – hermaphroditic part, FP – female part, MP – male part; Euphallics (E), Aphallics (A) and Hemiphallics: He (hemiphallic elongate), Hm (hemiphallic medium) and Hxs (hemiphallic extra small)

*Vertigo antivertigo* is a widespread Eurosiberian hygrophilous land snail inhabiting wetlands and fen habitats across most of Europe. It is a minute species (shell height up to 2.3 mm) and a simultaneous hermaphrodite exhibiting phally polymorphism, with euphallic, aphallic, and hemiphallic morphs co-occurring within populations (Fig. 1). Eggs are laid as single-celled zygotes following internal fertilization, embryogenesis lasts approximately 14 days at 20 °C, and sexual maturity is reached after about 45 days; life span rarely exceeds two years [21].

### Snail Origin

**Fig. 2:**
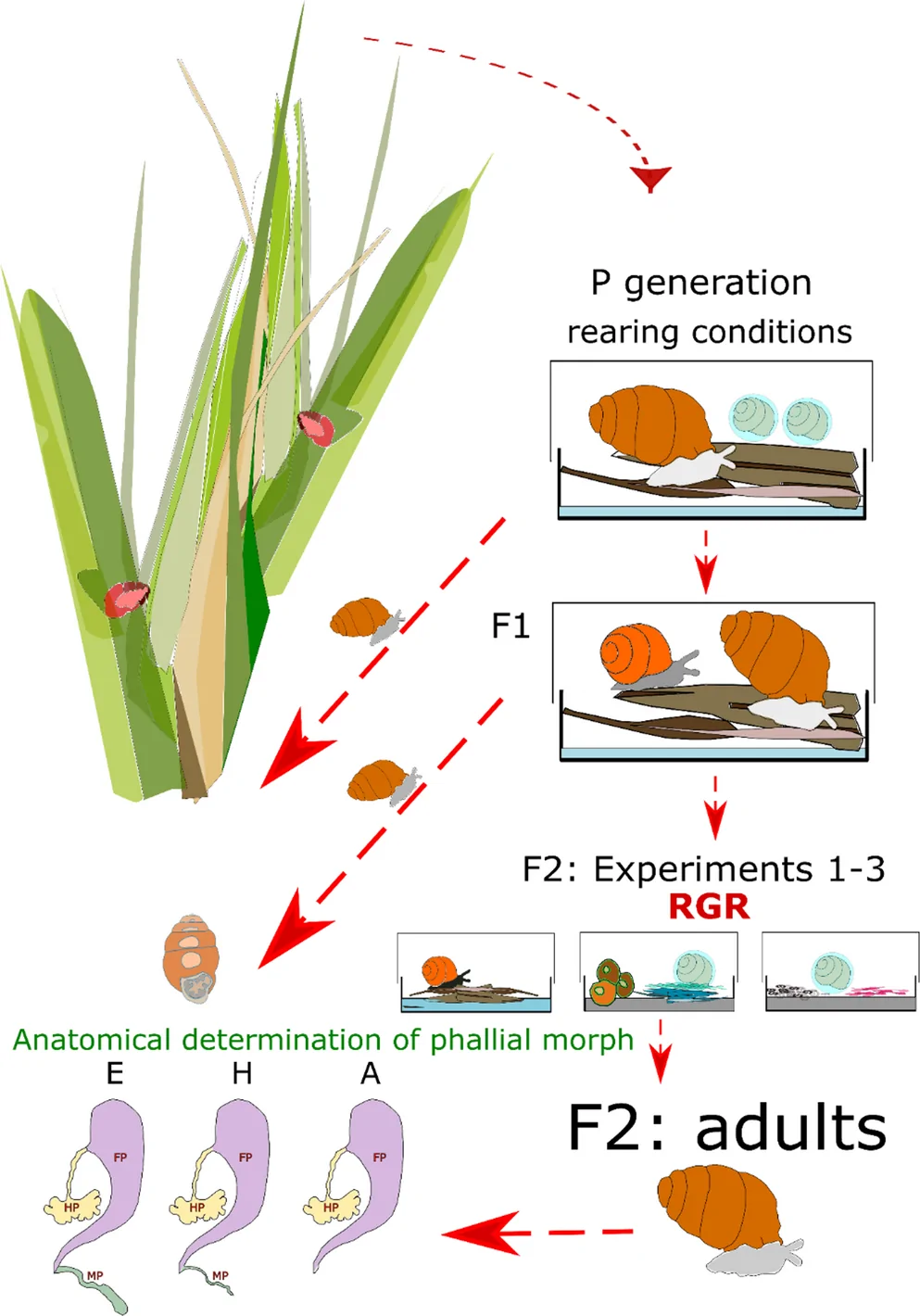
Overview of the study design, including field collection of snails, breeding cultures, experimental treatments, and subsequent measurements and anatomical analyses. Abbreviations: P – parental generation collected from the natural population; F1 – first laboratory generation; F2 – offspring of F1 used in experiments; RGR – relative growth rate; E – euphallic; H – hemiphallic; A – aphallic

Experimental individuals were obtained from laboratory breeding cultures established from snails collected at a single locality in Poland (52.42516, 16.77380) during 2021–2023. Field-collected snails (parental generation, P) were maintained individually under laboratory conditions. For experimental purposes, we used second-generation (F2) offspring produced exclusively via self-fertilization by virgin F1 individuals (Fig. 2), ensuring a known mating history and a standardized reproductive background across treatments.

### Microbial Culturing Protocols

All microorganisms were obtained from habitats in which *V. antivertigo* occurs. Most microbial inocula originated from the same field site as the snails (see Sect. 2.2), whereas the cyanobacterium *Limnothrix* sp. was isolated from a freshwater Przebędowo Reservoir (GPS: 52°35′11″ N, 17°0′56″ E), where *V. antivertigo* also occurs in nearby sedge and reed beds.

Fecal bacteria were obtained as mixed, cultured communities from snail feces. Material collected from multiple individuals was suspended in sterile water, plated on LB agar, and incubated. The resulting bacterial biomass was resuspended and used as a mixed inoculum, adjusted to defined optical densities for experimental application. Litter-associated bacteria were obtained from decaying plant material collected from the habitat, using the same culturing protocol as for fecal bacteria and pooling material from multiple samples. Fungal communities were isolated from litter and soil collected at the same field site (11 species; Supplementary Table S1; see also Supplementary Material, Section S1) and prepared as pooled inocula representing the most common taxa identified (see Supplementary Material, Section S1). The *Limnothrix* sp. strain used in this study was previously tested using multiple toxicological methods (enzyme-linked immunosorbent assays, noncompetitive time-resolved fluorescence immunoassay, high-performance liquid chromatography coupled with diode array UV detection, liquid chromatography coupled with mass spectrometry) and was found to be free of commonly screened cyanotoxins and bioactive metabolites including anabaenopeptins, anatoxin-a, cylindrospermopsin, microcystins, nodularin, and saxitoxins [22].

All microorganisms were cultured under laboratory conditions prior to experimental use. Detailed protocols for isolation, culturing, and preparation of all microorganisms are provided in the Supplementary Material (Section S1).

### Experimental Design

The study comprised three experimental series designated to examine how different microbial resource regimes influence early-life survival, growth and reproductive morphology (phally morph expression). Each experiment addressed a distinct component of these microbial resource regimes and is described separately below.

The experiments were conducted sequentially and were not intended as direct replicates. Instead, methodological adjustments were introduced between experiments based on insights gained from preceding treatments. To account for these differences, statistical analyses were performed separately for each experimental series.

Experiments were initiated either with early-stage juveniles (Experiment 1) or with eggs at an advanced developmental stage, with embryonic shells visible through the egg capsule (Experiments 2 and 3), representing stages that are particularly sensitive to environmental conditions. Experiments initiated from eggs ensured that individuals were exposed to microbial resource regimes immediately after hatching, i.e. from the earliest free-living stage.

As a reference treatment (Control) in all experiments, we used litter collected from the natural habitat of the study species (i.e. the site from which snails were collected; see Sect. 2.1) one day prior to use. After collection, litter was stored under cool (4 °C), moist conditions (relative humidity close to 100%) in closed containers and used within 24 h to preserve the naturally associated microorganisms, including litter-associated bacteria and fungi. A portion of this litter was sterilized to remove naturally associated microorganisms and used in specific treatments (see descriptions of individual experiments below).

Throughout all experimental series, snails were reared individually under controlled laboratory conditions (17 °C; 12 h:12 h light: dark photoperiod), each in a Petri dish (Ø 50 mm) lined with either cotton pads (standard procedure for breeding minute land snails; [23, 24]) or a plaster substrate, depending on the experiment. Microbial resources were applied onto small filter paper discs (5 mm in diameter) placed within each dish, and individuals were positioned in its direct proximity to facilitate contact and feeding. Individuals were inspected at 7–10-day intervals. Detailed descriptions of rearing substrates, microbial delivery, and maintenance procedures are provided in the Supplementary Material (Section S2).

Experimental treatments were coded according to their components (C – cyanobacteria, LB – litter bacteria, FB – fecal bacteria, F – fungi, BL – blended litter, LPL – large-piece litter, SL – sterilized litter). In all series, large-piece litter (LPL) served as the Control. Treatment composition and sample sizes are summarized in Table 1. All experimental treatments were conducted exclusively on F2 individuals. A detailed description of experimental procedures is provided in the Supplementary Material (Section S3).

**Table 1:** Summary of treatments applied in Experiments 1–3 (Exp. 1–3). *n* denotes the number of individuals (each reared separately and treated as an independent experimental unit). Table cells indicate either the presence of a given component (for solid substrates: LPL – large-piece litter (Control), SL – sterilized litter, BL – blended litter) or the volume of microbial suspension applied (µl) for microbial treatments (FB – fecal bacteria: FB1: OD 0.01; FB2: OD 0.1, LB – litter bacteria, C – cyanobacteria, F – fungi); “–” indicates absence

	Treatment	n	LPL	SL	FB	LB	C	F	BL
Exp. 1	SL	32	–	YES	–	–	–	–	–
	SL+FB1	32	–	YES	20 µl	–	–	–	–
	SL+FB2	31	–	YES	20 µl	–	–	–	–
	Control	32	YES	–	–	–	–	–	–
Exp. 2	C	30	–	–	–	–	350 µl	–	–
	LB	29	–	–	–	20 µl	–	–	–
	C + LB	19	–	–	–	20 µl	330 µl	–	–
	C + LB+SL	17	–	YES	–	20 µl	330 µl	–	–
	C + LB+FB	18	–	–	10 µl	10 µl	330 µl	–	–
	Control	29	YES	–	–	–	–	–	–
Exp. 3	F	30	–	–	–	–	–	350 µl	–
	BL	28	–	–	–	–	–	–	YES
	F + BL	30	–	–	–	–	–	350 µl	YES
	Control	30	YES	–	–	–	–	–	–

#### Experiment 1 - Bacterial Supplementation Under Sterile Litter Conditions

Experiment 1 was designed to test whether feces-derived bacteria can compensate for the absence of litter-associated microorganisms and thereby mitigate early-life growth suppression and mortality. The experiment was initiated with early-stage juveniles.

Snails were reared individually on cotton pads. Treatments consisted of sterilized litter (SL – litter devoid of naturally occurring microorganisms) with or without supplementation of *Vertigo antivertigo* fecal bacteria, while fresh large-piece litter (LPL) served as the Control (Table 1). Two concentrations were tested (FB1 and FB2), corresponding to different optical densities (OD 0.01 and 0.1) of the fecal bacterial suspensions.

#### Experiment 2 - Effects of a Cyanobacterium and Bacterial Interactions On Early Development

Experiment 2 was designed to assess the effects of a cyanobacterium on early-life survival, growth, and development, and to test whether these effects were modified by co-occurring microbial resource regimes. The experiment was initiated with eggs at an advanced developmental stage (with visible embryonic shells).

Snails were reared individually on plaster substrate to reduce unintended ingestion of substrate material. Treatments included: cyanobacteria alone (C), litter-associated bacteria alone (LB; OD 0.1), a combination of cyanobacteria and litter-associated bacteria (C + LB), and their combinations with either sterilized litter devoid of naturally associated bacteria (C + LB+SL) or additional fecal bacteria (C + LB+FB; OD 0.1) (Table 1).

#### Experiment 3 - Fungal Supplementation and Substrate Structure Effects

Experiment 3 examined the role of fungal supplementation in shaping survival, growth, and reproductive morphology (phally morph), and assessed its effects both independently and in combination with structurally modified litter, relative to intact litter containing its natural microbial communities. As in Experiment 2, the experiment was initiated with advanced-stage eggs.

To reduce spatial heterogeneity in food distribution, blended litter was introduced as a more homogeneous food resource, while plaster continued to serve as the rearing substrate. Treatments included fungi alone (F), blended litter alone (BL), their combination (F + BL), and a large-piece litter (LPL) as a Control (Table 1). This design allowed us to assess the role of fungi both independently and in combination with structurally modified litter, compared with intact litter containing its natural microbial communities.

### Measurements and Anatomy

Because our aim was to compare relative developmental traits under contrasting microbial resource regimes within a single species, rather than absolute growth rates across taxa, shell growth was quantified as the increase in the number of shell whorls, counted according to Janssen [25].

Relative growth rate (RGR) was calculated for each individual following Hoffmann and Poorter [26]:

$$\:\mathrm{R}\mathrm{G}\mathrm{R}=\:\frac{\mathrm{ln}\left({\mathrm{S}}_{2}\right)-\mathrm{l}\mathrm{n}\left({\mathrm{S}}_{1}\right)}{{\mathrm{t}}_{2}-\:{\mathrm{t}}_{1}}$$

where $\:{S}_{1}$ and $\:{S}_{2}$represent shell size (number of whorls) at the first and last observation, respectively, and $\:{t}_{1}$and $\:{t}_{2}$denote the corresponding observation times. The final observation ($\:{t}_{2}$) corresponded either to the day of death or to the attainment of final shell size.

Anatomical studies were conducted on F1 individuals and on all F2 individuals that survived until the end of the experimental treatments. F1 individuals were not subjected to experimental treatments but were included as a reference group to enable comparisons of reproductive morphology across generations. Following reproduction, these individuals were preserved in 96% ethanol. In addition, in 2021, a reference sample from the founding population (P) was collected and examined.

In Experiments 1 and 2, hemiphallic individuals were treated as a single category (H). However, owing to the pronounced morphological variation in hemiphallic penes, a more detailed subdivision of hemiphallic morphs was introduced in Experiment 3 following Pokryszko [10]. Hemiphallic individuals were classified into three categories: He (hemiphallic elongate), Hm (hemiphallic medium), and Hxs (hemiphallic extra small) (Fig. 1).

### Statistical Methods

Growth analyses were conducted separately for each experimental series and included all individuals assigned to a treatment, regardless of whether they survived to sexual maturity. For each individual, RGR was calculated from the first observation to either the day of death or the achievement of final size (maximum duration of 140 days). Therefore, early mortality shortened the observation period rather than creating missing growth data. Treatment-specific sample sizes for growth analyses are provided in Table 1.The data set was initially examined for normality using the Shapiro-Wilk test and for homogeneity of variances with Levene’s test. When parametric assumptions were considered acceptable, one-way ANOVA was applied to compare group means, followed by Tukey’s HSD post hoc test for pairwise comparisons. When variance heterogeneity was detected, Welch’s ANOVA was used instead, followed by Games-Howell post hoc test.

Survival was analysed separately for each experimental series *(survival* and *survminer* package). Time-to-event was defined as the number of days from the start of observation to death or reaching the final size. Individuals that reached final size before death were treated as censored observations, while death was considered the event. The Kaplan-Meier survival curves were compared across treatments using log-rank tests, and Cox proportional hazards models were employed to estimate hazard ratios (HRs) with 95% confidence intervals (see Figs. S1 – S3). The proportional hazards assumption was tested with Schoenfeld residuals. These analyses were performed in R (version 4.4.2).

Differences in the distribution of phally morphs among generations (P, F1, F2) and treatments were analysed using chi-squared tests, performed in PAST [27].

## Results

### Overview of Experimental Outcomes and Survival

A total of 480 snails were included in the study. Of these, 387 individuals from the F2 generation were used in experiments assessing relative growth rate (RGR). Direct inspection confirmed that the snails fed on the provided microbial resources, as evidenced by food-filled intestines and the presence of feces.

Across all experimental series, juvenile mortality was high. Only 65 experimental snails (F2 generation) survived to attain final shell size and sexual maturity, allowing determination of phally morph in 64 individuals (Table 2). In addition, 51 individuals from the natural parental population (P) and 24 individuals from the F1 generation were dissected for comparative analyses. F1 individuals were included only when at least some F2 offspring from the corresponding treatment survived, ensuring that intergenerational comparisons reflected lineages that successfully passed early-life filters.

**Table 2:** Results of dissections and survival in Experiments 1–3. *n* denotes the total number of individuals; *S* indicates individuals that survived to the adult stage. Generations are defined as follows: P – wild-caught individuals; F1 – offspring of P; F2 – offspring of F1. All experimental treatments were conducted exclusively on F2 individuals, whereas F1 individuals are included for comparative purposes only. The generation used in the experiments is shown in **bold**. Morphological categories are defined as: E – euphallic; A – aphallic; H – hemiphallic, with subcategories distinguished in Experiment 3 (He – elongate; Hm – medium; Hxs – extra-small). Treatments are abbreviated as follows: SL – sterilized litter; FB – fecal bacteria (FB1 – OD = 0.01; FB2 – OD = 0.1); C – cyanobacteria; LB – litter-associated bacteria; F – fungi; BL – blended litter; Control – large pieces of litter (LPL)

	Gen.	Treatment	n	S	E	A	H	He	Hm	Hxs
Experiment 1	P		51	n/a	52	0	9			
	F1		24	n/a	6	7	11			
	**F2**	**SL**	32	0	0	0	0			
	**F2**	**SL+FB1**	32	0	0	0	0			
	**F2**	**SL+FB2**	31	4	4	0	0			
	**F2**	**Control**	32	28	11	3	13			
Experiment 2	**F2**	**C**	30	0	0	0	0			
	**F2**	**LB**	29	0	0	0	0			
	**F2**	**C + LB**	19	0	0	0	0			
	**F2**	**C + LB+SL**	17	0	0	0	0			
	**F2**	**C + LB+FB**	18	0	0	0	0			
	**F2**	**Control**	29	13	0	3	10			
Experiment 3	F1	F + BL	7	n/a	7	0	0	0	0	0
	F1	Control	11	n/a	8	0	3	3	0	0
	**F2**	**F**	30	0	0	0	0	0	0	0
	**F2**	**BL**	28	0	0	0	0	0	0	0
	**F2**	**F + BL**	30	11	0	6	5	0	1	4
	**F2**	**Control**	30	9	0	4	5	0	3	2

### Experiment 1 – Bacterial Supplementation Under Sterile Litter Conditions

A total of 127 juvenile snails were included in Experiment 1. Relative growth rate differed significantly among treatments (F₃,₁₂₂ = 21.1, *p* < 0.001). The highest mean RGR was observed in the Control treatment with fresh large-piece litter (0.024 ± 0.009 whorl day⁻¹), whereas the lowest was recorded under sterilized litter alone (0.007 ± 0.005 whorl day⁻¹).

**Fig. 3:**
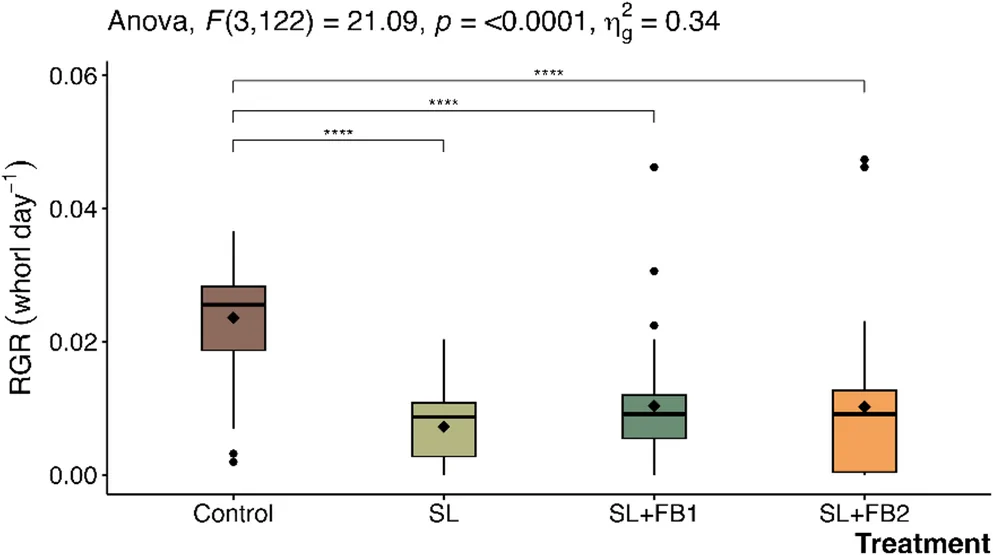
Snails relative growth rate (RGR) across different treatments in Experiment 1: SL – sterilized litter, FB – faeces bacteria (FB1-OD = 0.01; FB2-OD = 0.1). The box plots represent the median (line), the interquartile range (25th – 75th percentiles), and the 10th and 90th percentiles (whiskers). Black diamonds indicate mean values. Statistical comparisons were conducted using ANOVA with significant post hoc differences indicated by asterisks (**** *p* < 0.0001)

All treatments involving sterilized litter resulted in significantly lower RGR values compared to the Control, with reductions of 71% in the SL treatment (*p* < 0.001) and 58% in both SL+FB1 and SL+FB2 treatments (*p* < 0.001; Fig. 3). No significant differences were detected among the sterilized-litter treatments themselves.

Survival also varied significantly among treatments (log-rank test: χ^2^ = 19.3, df = 3, *p* < 0.001). Median survival was not reached in the Control group, while it was 76 days for the SL treatment, 63 days for SL+FB1, and 76 days for SL+FB2. Compared to the Control, the hazard of death was significantly higher in the SL treatment (HR = 6.79, 95% CI: 2.07–22.31, *p* < 0.01), SL+FB1 (HR = 8.21, 95% CI: 2.50–26.91, *p* < 0.001), and SL+FB2 (HR = 4.50, 95% CI: 1.34–15.11, *p* < 0.05).

**Fig. 4:**
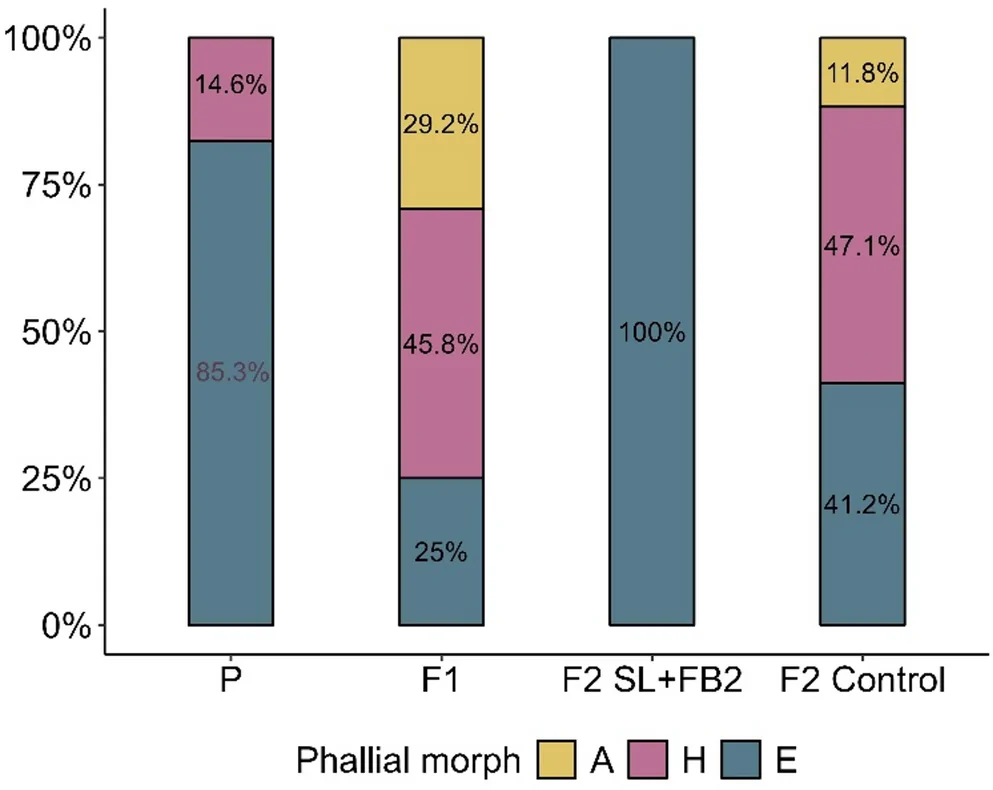
Proportions of different morphs in Experiment 1 across P, F1, and F2 employed generations. Morph types are as follows; A – aphallics; H – hemiphallics; E – euphallics

Only 32 individuals survived to maturity in Experiment 1 (Table 2), allowing assessment of phally morph in 31 snails (one shell had been accidentally destroyed): 27 from the Control group and 4 from the SL+FB2 treatment. Comparison of morph distributions among wild (P), F1, and F2 generations revealed significant differences (χ^2^ = 173.81, df = 6, *p* < 0.001). The natural population was dominated by euphallic individuals (82.35%), whereas the F1 generation showed a higher proportion of hemiphallic (45.8%) and aphallic (29%) morphs. The F2 Control group displayed a more even morph distribution, while all surviving individuals in the SL+FB2 treatment were euphallic (Fig. 4).

### Experiment 2 – Effects of a Cyanobacterium and Bacterial Interactions On Early Development

**Fig. 5:**
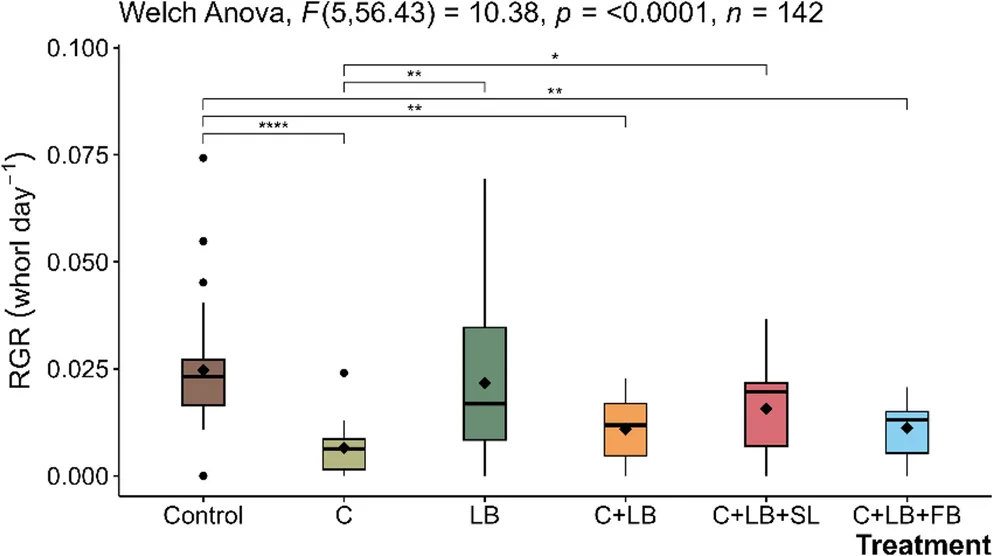
Snails relative growth rate (RGR) across different treatments in Experiment 2: C – cyanobacteria; LB – litter bacteria; FB – faeces bacteria; SL – sterilized litter. The box plots represent the median (line), the interquartile range (25^th^– 75^th^ percentiles), and the 10^th^ and 90^th^ percentiles (whiskers). Black diamonds indicate mean values. Statistical comparisons were performed using Welch’s ANOVA with significant post hoc differences indicated by asterisks (* *p* < 0.05, (** *p* < 0.01, **** *p* < 0.0001)

In total, 142 snails were included in Experiment 2, and their RGR was assessed. The highest mean RGR value was recorded in the Control group (0.025 ± 0.016 whorl day^− 1^), whereas the lowest was observed in the group exposed to a cyanobacterium (0.006 ± 0.005 whorl day^− 1^). The analysis revealed significant differences in RGR values among the treatments studied (F_5, 56.4_ = 10.4, *p* < 0.001). Post-hoc comparisons showed that the Control group had significantly higher RGR values compared to the C treatment (−74%; *p* < 0.001), the C + LB treatment (−56%; *p* < 0.01), and the C + LB+FB treatment (−54%; *p* < 0.01). Additionally, significant differences were also observed between the C group and the LB group (+ 236%; *p* < 0.01). A difference was also observed between the C treatment and the C + LB+SL treatment (+ 141%; *p* < 0.05) (Fig. 5).

Survival varied significantly among treatments in Experiment 2 (log-rank test: χ^2^ = 78.4, df = 5, *p* < 0.001). Only the Control group included individuals that reached maturity (13/29), while all other treatments experienced complete mortality. Compared to the Control, all experimental treatments had a significantly higher hazard of death in the Cox model (HR = 6.98–32.56, all *p* < 0.001). However, since the proportional hazards assumption was violated (global test: *p* < 0.001), these hazard ratios should be interpreted cautiously as average effects over the observation period.

Mortality was absolute: none of the F2 individuals in the experimental treatments survived to maturity, whereas 13 F2 individuals survived in the Control group (Table 2). As a result, reproductive morphology could not be assessed in the experimental treatments, and no comparisons of phally morph distributions among treatments or generations were possible for this experiment.

### Experiment 3 – Fungal Supplementation and Substrate Structure Effects

**Fig. 6:**
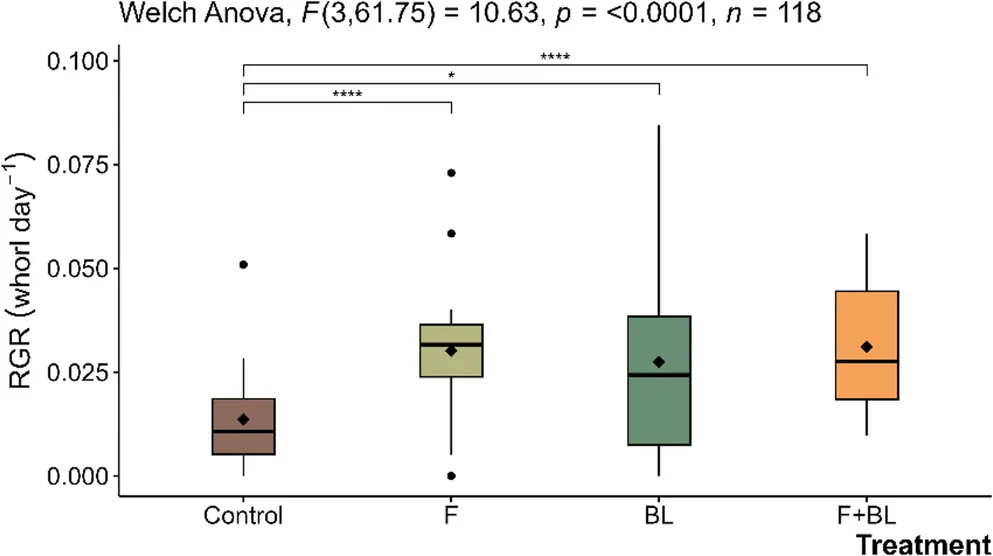
Snails relative growth rate (RGR) across different treatments in Experiment 3: F - fungi; BL - blended litter. The box plots represent the median (line), the interquartile range (25th – 75th percentiles), and the 10th and 90th percentiles (whiskers). Black diamonds indicate mean values. Statistical comparisons were made using Welch’s ANOVA with significant post hoc differences indicated by asterisks (* *p* < 0.05, **** *p* < 0.0001)

In total, we used 118 snails for Experiment 3, for which the RGR was assessed. The highest mean RGR value was recorded in the F + BL group (0.031 ± 0.015 whorl day^− 1^), while the lowest was observed in the Control group (0.014 ± 0.013 whorl day^− 1^). The analysis revealed significant differences in RGR values among the studied groups (F_3,61.75_ = 10.6, *p* < 0.001). Post hoc analysis indicated that the Control group exhibited significantly lower RGR values compared to all other treatments, with reductions of 54% compared to the F treatment (*p* < 0.001), 50% compared to the BL treatment (*p* < 0.01), and 55% relative to the F + BL treatment (*p* < 0.001). This indicated that the applied treatments positively influenced the RGR relative to the Control condition. No significant differences were observed between the F, BL, and F + BL groups, indicating comparable effects of these treatments on the RGR (Fig. 6).

Survival varied significantly among treatments in this experiment (log-rank test: χ^2^ = 46.9, df = 3, *p* < 0.001). Only individuals in the Control and F + BL treatments survived to maturity (9/30 and 11/30, respectively), while complete mortality occurred in the F and BL treatments. Compared to the Control, the risk of death was significantly higher in the F treatment (HR = 2.33, 95% CI: 1.29–4.22, *p* < 0.01) and in the BL treatment (HR = 5.08, 95% CI: 2.65–9.73, *p* < 0.001). The F + BL treatment did not differ significantly from the Control (HR = 0.54, 95% CI: 0.29–1.03, *p* = 0.06). However, these hazard ratios should be interpreted cautiously since the proportional hazards assumption was violated (global test: *p* < 0.001).

Phally morph distributions differed significantly among generations and treatments (χ^2^ = 496.27, df = 12, *p* < 0.001). The F1 Control generation was dominated by euphallic (E) individuals (72.73%), with the remaining 27.27% classified as hemiphallic-elongate (He), the hemiphallic morph with the largest penis; no individuals of other morph categories were observed. In contrast, the F2 Control generation exhibited a more even distribution of phally morphs, with 44.44% classified as aphallic (A), 22.22% as hemiphallic-extra small (Hxs), and 33.33% as hemiphallic-medium (Hm).

**Fig. 7:**
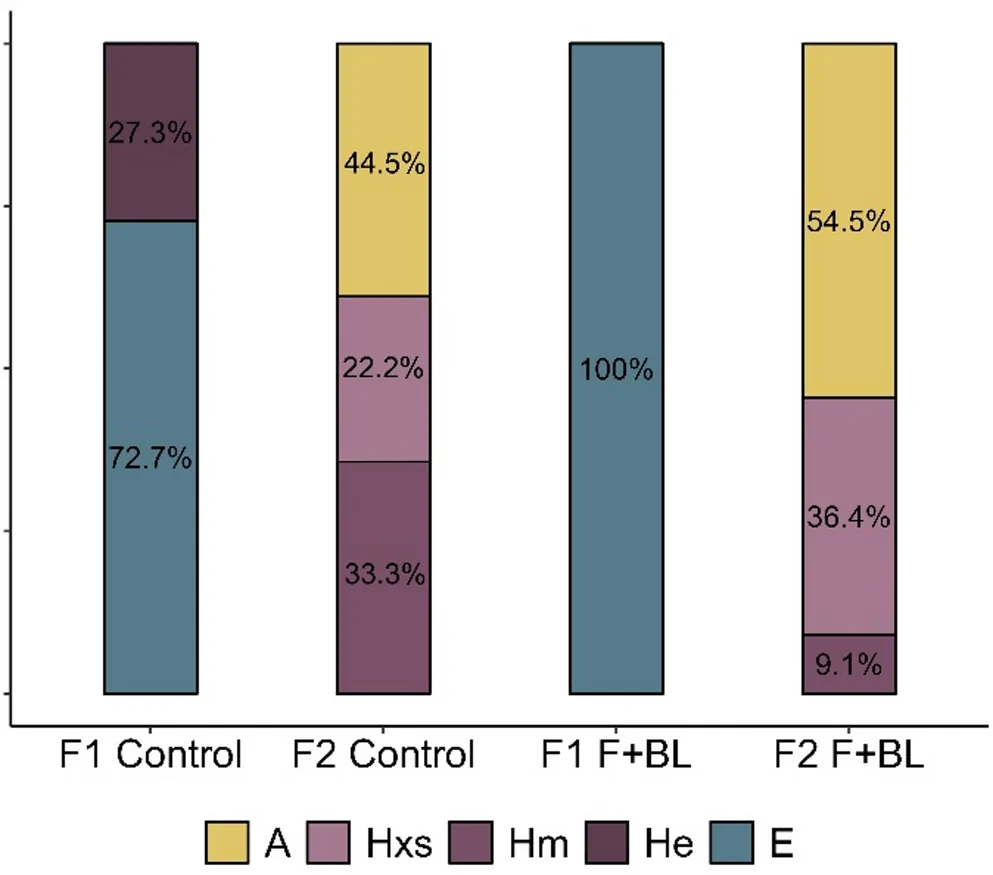
Proportions of different morphs in Experiment 3 across F1, and F2 employed generations. Morph types are as follows; A – aphallics; H – hemiphallics; Hxs – extra small hemiphalic; Hm – hemiphallic medium; He – hemiphallic elongate; E – euphallics

Within the F + BL treatment, all F1 individuals were euphallic (100%). However, F2 individuals in the same treatment showed a markedly different pattern, with the highest proportion classified as aphallic (54.55%), followed by Hxs (36.36%) and Hm (9.09%); no individuals were classified as He or E (Fig. 7).

## Discussion

### Microbial Resource Regimes As Early-life Ecological Filters

This study examined how microbial resource regimes naturally occurring in wetland habitats shape early-life survival, growth, and reproductive morphology in a terrestrial invertebrate. To assess the role of these regimes, we experimentally manipulated microorganisms to which individuals were exposed from the egg and juvenile stages that are characterized by high mortality and strong selective filtering in many invertebrates [5]. Our study demonstrates that early-life survival appears to be actively shaped by microbial resource regimes, with potential downstream consequences for the distribution of phally morphs within populations. Given the ecological roles of land snails in nutrient cycling and trophic interactions in wetlands [28–30], microbially mediated survival filtering during early ontogeny may contribute to variation in population structure under natural conditions. To our knowledge, this study provides the first experimental evidence that cyanobacteria can act as strong negative developmental filters in a terrestrial wetland invertebrate.

Across all experimental series, survival was the most consistent and biologically informative response variable, indicating that microbial resource regimes operate primarily as early-life ecological filters rather than merely modulating growth rates. While previous studies on land snail development have emphasized abiotic factors such as temperature, humidity, and calcium availability [6], our findings identify microbial resource regimes as integral components of the developmental environment.

### Bacterial Effects Under Sterile Conditions

Fecal bacteria of *V. antivertigo* were selected because they are naturally abundant in the species’ microhabitats and may represent nutritionally accessible resources [4]. However, bacterial supplementation alone did not compensate for the absence of intact litter-associated microorganisms. Although individuals receiving bacterial supplementation grew faster than those maintained on sterilized litter alone, devoid of naturally occurring microorganisms, survival remained extremely low, suggesting that bacterial addition provided only limited nutritional or developmental support under sterile substrate conditions. The applied bacterial dose may also have been insufficient to sustain normal development.

Survival was low across all treatments; however, among the few surviving individuals in bacterial treatments, only euphallic morphs were observed. Given the very small sample sizes, this pattern must be interpreted cautiously. Nonetheless, previous observations in *Carychium tridentatum* suggest that feces-derived microbial components can influence early development in minute land snails [23]. Our results therefore indicate that bacteria alone are unlikely to substitute for complex litter-associated microorganisms in supporting early-life development.

### Cyanobacteria as Strong Negative Developmental Filters

Experiment 2 revealed the cyanobacterium *Limnothrix* sp.) as a strong negative developmental filter. Cyanobacterial exposure suppressed growth and resulted in complete juvenile mortality, irrespective of bacterial supplementation. Cyanobacterial toxicity has been documented primarily in vertebrates [16–19], whereas our findings suggest that these concerns may also apply to terrestrial invertebrates associated with wetland habitats.

Although the *Limnothrix* sp. strain used in this study was previously reported to lack commonly studied cyanotoxins [22], the negative effects observed in the present study suggest that this strain may synthesize other, as yet unidentified, bioactive compounds that exert harmful effects on aquatic biota. Consistent with this suspicion, filtered extracts of this strain were recently shown to reduce the survival of two filter-feeding cladocerans, *Daphnia magna* and *Daphnia pulicaria*, by more than 50% [22]. Another feature of this strain, revealed in that previous work, is its high potential to produce extracellular polymeric substances (EPS), which make its biomass noticeably mucilaginous. Perhaps, these substances, when produced in excess, may mechanically interfere with snail feeding and thereby negatively affect life history traits of *V. antivertigo*. However, EPS may also interact with and absorb harmful cyanobacterial metabolites present in water [31, 32], thereby facilitating their transfer to snails during feeding and contributing to harmful effects. Cyanobacteria are known to produce a wide range of bioactive secondary metabolites beyond the most commonly screened toxins, some of which may affect invertebrate physiology [33]. In addition, cyanobacterial biomass may be of low nutritional quality or may interfere with digestion, particularly in early developmental stages [34]. Cyanobacteria can also influence surrounding microorganisms and associated ecological interactions [35], potentially disrupting beneficial interactions between microorganisms associated with litter. These mechanisms, acting alone or in combination, may explain the strong negative developmental effects observed in this study.

Given the increasing prevalence of cyanobacterial blooms driven by eutrophication and climate-related changes in hydrology and temperature [36], exposure to cyanobacteria may represent an underappreciated constraint on the early development of wetland-associated terrestrial fauna. The observed survival collapse indicates that cyanobacteria can substantially alter early-life filtering dynamics in the studied invertebrate. Further studies on this strain are needed to better understand and explain the mechanisms underlying its apparent toxicity.

### Fungi as Nutritional and Developmental Modulators

Fungal supplementation combined with blended litter supported the highest survival and growth rates among the microbial exposure treatments in Experiment 3 and was the only option (aside from the Control) that allowed completion of the life cycle. These results are consistent with earlier suggestions that *Vertigo* species utilize fungi as an important food resource [15], indicating that fungi can function as key nutritional and developmental components. However, fungal effects were strongly context-dependent. Fungi alone, as well as blended litter alone, resulted in complete mortality of *V. antivertigo*, whereas large-piece litter (Control) sustained survival without supplementation. This pattern suggests that microbial resource regimes and substrate structure jointly determine developmental outcomes.

Fungal treatments were also associated with shifts in reproductive morphology, including increased proportions of aphallic and hemiphallic individuals with reduced copulatory structures relative to Controls (Table 2). Although mortality limits firm conclusions, such shifts are consistent with the possibility that microbial resource regimes influence reproductive development early in ontogeny. The fungal assemblage included common soil and litter taxa such as *Clonostachys rosea* and *Trichoderma harzianum*, species known for diverse ecological interactions [37–43]. Notably, *T. harzianum* has been reported to induce molluscicidal effects and histopathological alterations in other gastropods [44, 45]. While we did not assess tissue-level pathology, these reports indicate that some fungal taxa can interfere with normal physiological and reproductive processes in snails.

### From Laboratory Bottlenecks to Life-history Interpretation

Several limitations inherent to laboratory experiments must be acknowledged. The experimental series differed in substrates and microbial delivery as protocols were refined over time, which precludes direct quantitative comparisons among experiments. Accordingly, our findings should be interpreted as identifying broad ecological patterns rather than providing definitive explanations.

While treatments were defined by controlled addition or removal of specific microbial components, we did not directly quantify microbial composition across treatments. Nevertheless, the experimental design relied on established manipulations (e.g. sterilized vs. non-sterilized litter, defined inocula) expected to generate contrasting microbial resource regimes. Future studies should incorporate microbiological or molecular approaches to validate these differences.

All experimental treatments were conducted on F2 individuals derived from repeated self-fertilization, which raises the possibility that inbreeding effects may have contributed to the observed differences between generations. Vertiginidae are known to frequently self-fertilize, and copulation has rarely been observed under laboratory conditions, suggesting that selfing may be common [24]. However, quantitative data on selfing rates in natural populations remain limited. Although inbreeding depression cannot be excluded, we consider it more likely that the observed differences between F1 and F2 generations reflect the influence of laboratory conditions. Early developmental stages in these minute land snails are highly sensitive to environmental conditions, and the simplified and experimentally manipulated microbial resource regimes used here may have amplified developmental differences between generations.

Higher mortality in experiments initiated with eggs compared to juveniles indicates increased vulnerability during early-developmental stages [9]. Laboratory observations suggest that most eggs survived to hatching, whereas mortality occurred predominantly after hatching. A substantial proportion of mortality therefore likely took place shortly after hatching, when individuals begin feeding and are directly exposed to experimental conditions. However, because observations were conducted at discrete time intervals, it was not always possible to distinguish precisely between immediate post-hatching mortality and later early juvenile mortality. While microbial resources used in the experiments are unlikely to directly influence embryonic survival prior to hatching, several factors may contribute to high early mortality under laboratory conditions. These include sensitivity to microenvironmental conditions, such as moisture, absence of natural substrate complexity, and potential disruptions of beneficial microbial interactions. Handling procedures and laboratory conditions may also have contributed to increased mortality during early ontogeny. Although the specific drivers of mortality differ between laboratory and natural settings, early life stages in natural populations also experience substantial mortality due to predation, desiccation, and environmental variability [6]. The survival observed here is therefore likely to reflect biologically realistic sensitivities, although laboratory conditions inevitably simplify environmental complexity.

High mortality across treatments limited our ability to draw unequivocal conclusions regarding the effects of microorganisms on sexual morph expression. Nevertheless, the observed patterns suggest that microbial resource regimes experienced early in ontogeny may influence reproductive morphology. Any individual-level link between growth rate and phally morph should be interpreted cautiously because phally morph could only be assigned to individuals that survive to maturity, while growth rate was calculated for all individuals. Consequently, formal tests of a growth–morph relationship in this dataset would depend on survival and might reflect selective survival across treatments rather than a direct developmental connection. The recurrent shifts in phally morph distributions under specific microbial resource regimes indicate that this question warrants further investigation under experimental designs that allow higher replication and longer-term tracking of survivors.

Finally, nutritional and environmental effects cannot be fully disentangled, but the presence of food-filled digestive tracts confirms active consumption of provided resources. Notably, aphallic individuals were absent in the parental population but appeared in laboratory-reared generations (F1 and F2). This pattern suggests that factors associated with laboratory conditions, such as simplified environmental structure, altered microbial resource regimes, or repeated self-fertilization, may influence morph expression independently of experimental treatments. Early-life survival may further contribute to this pattern by selectively filtering individuals, thereby altering morph frequencies among survivors.

### Conclusions

Our results support the hypothesis that intact, naturally derived microbial resource regimes associated with fresh litter play a key role in shaping early-life survival and growth in *Vertigo antivertigo*. Access to such complex microbial resource regimes resulted in higher survival and faster growth. One exception was the treatment combining fungi with blended litter, suggesting that fungi constitute a key component supporting growth and survival in this species. In most cases, however, simplified or disrupted microbial regimes led to reduced survival and growth.

Microorganisms respond rapidly to environmental change, including eutrophication, hydrological alteration, and climate-driven shifts in temperature and moisture [46, 47]. Variation in microbial resource regimes may therefore modify the strength and selectivity of early-life survival. Our findings indicate that such filtering can influence not only which individuals reach maturity but also the distribution of reproductive morphs within populations of a phally polymorphic species, with potential consequences for genetic diversity and population persistence [14]. Microbial resource regimes encountered early in development may thus represent an underrecognized driver of life-history and reproductive variation in terrestrial invertebrates.

## Supplementary Information

Below is the link to the electronic supplementary material.


Supplementary Material 1


## Data Availability

The datasets generated during and/or analysed during the current study are available from the corresponding author on reasonable request.
