# Supplementary material for: Microbial Resource Regimes Shape Early Survival, Growth, and Reproductive Morphology in a Phally Polymorphic Terrestrial Gastropod
Source: Microb Ecol. 2026 Jun 19;89:166. doi: 10.1007/s00248-026-02814-2 (PMC13526737; doi:10.1007/s00248-026-02814-2)
Supplement: Supplementary file 1 [file 248_2026_2814_MOESM1_ESM.docx]

**Supplementary Materials**

**Microbial resource regimes shape early survival, growth, and reproductive morphology in a terrestrial gastropod with phally polymorphism**

Zofia Książkiewicz^1^, Karolina Górzyńska^2^, Ewa Kosicka^3^, Łukasz Wejnerowski^4^, Michał Rybak^5^

^1^ Department of General Zoology, Faculty of Biology, Adam Mickiewicz University, Poznań, Uniwersytetu Poznańskiego 6, 61–614 Poznań, Poland, email: [zofksi@amu.edu.pl](mailto:zofksi@amu.edu.pl)

^2^ Department of Systematic and Environmental Botany, Faculty of Biology, Adam Mickiewicz University, Poznań, Poland; e-mail: karjan@amu.edu.pl,

^3^ Department of Bioenergetics, Faculty of Biology, Adam Mickiewicz University in Poznań, Uniwersytetu Poznańskiego 6, 61-614, Poznań, Poland, e-mail: ewa.kosicka@amu.edu.pl

^4^ Department of Hydrobiology, Institute of Environmental Biology, Faculty of Biology, Adam Mickiewicz University, Uniwersytetu Poznańskiego 6, 61-614, Poznań, Poland, e-mail: wejner@amu.edu.pl

^5^Department of Water Protection, Faculty of Biology, Adam Mickiewicz University, Poznań, Uniwersytetu Poznańskiego 6, 61–614 Poznań, Poland, e-mail: m.rybak@amu.edu.pl

Corresponding Author: Zofia Książkiewicz, e-mail: [zofksi@amu.edu.pl](mailto:zofksi@amu.edu.pl)

**Materials and Methods**

**S1. Microbial culturing protocols**

**Bacteria**

Bacterial cultures were prepared from two sources: snail feces and litter collected from the snail habitat. Fecal bacteria were obtained from five snails collected at the culture origin site. Each snail was kept individually in a sterile Falcon tube at 17 °C. After approximately 24 h, snails were removed, and feces were collected aseptically under a laminar-flow hood.

Depending on the target bacterial group, feces alone or a mixture of feces and litter were suspended in sterile water and thoroughly vortexed. A 100 µl aliquot of the suspension was plated on Luria–Bertani (LB) agar and incubated overnight at 37 °C.

Litter-associated bacteria were obtained using the same procedure. Litter was immersed in distilled water for 30 min and vortexed for 30 s, and bacteria were cultured directly from the resulting suspension.

For experimental use, 5 ml of sterile water was added to LB plates containing bacterial growth and shaken at 100 rpm for 10 min. The resulting liquid culture was collected and diluted to optical densities (OD) of 0.01 or 0.1, depending on the experimental treatment.

**Cyanobacteria**

The filamentous cyanobacterium *Limnothrix* sp. strain W3 was used. This strain is free of commonly studied cyanotoxins (anatoxin-a, anabaenopeptins, cylindrospermopsin, saxitoxin, microcystins, and nodularin). It was isolated in 2020 from phytoplankton biomass collected from the Przebędowo Reservoir (52.58639 N, 17.01556 E) using the serial dilution technique.

Stock cultures were maintained in 250-ml glass Erlenmeyer flasks containing Wright–Chu (WC) medium and incubated in a Fito700 phytotron chamber (Biogenet, Poland) at 20 ± 0.5 °C, a light intensity of 48 µmol photons m⁻² s⁻¹, and a 16 h:8 h light:dark photoperiod. Cultures were refreshed every three weeks. Two-week-old biomass was used for snail experiments.

**Fungal isolation, identification, and preparation**

Fungal isolation and identification were performed using soil and litter samples collected in 2024 from the locality inhabited by the study species. Samples were stored in labeled bags under cool conditions until processing.

Soil-borne fungi were isolated using the serial dilution method [1]. One gram of soil was suspended in 10 ml of sterile double-distilled water (10⁻¹), and serial dilutions ranging from 10⁻⁴ to 10⁻⁶ were prepared. Aliquots (1.0 ml) from each dilution were plated on Potato Dextrose Agar (PDA; A&A Biotechnology, Poland) supplemented with chloramphenicol (100 mg L⁻¹) and incubated at 25 °C.

Litter-associated fungi were isolated by examining litter fragments under a stereomicroscope (Olympus SZ61-TR), followed by transfer of visible mycelium to PDA plates. All plates were incubated at 25 °C and monitored daily. Emerging colonies were subcultured onto fresh PDA plates and incubated for seven days.

Isolates were identified using the molecular method. DNA was isolated using a Quick-DNA Fungal/Bacterial Miniprep Kit (Zymo Research, USA) according to the manufacturer’s protocol and stored at -20 ° C. A three pair of primers were employed to amplify regions ITS1F [2] and ITS4 [3], that anneal to the ribosomal cassette, which consisted of SSU (partial), ITS1, 5.8S, ITS2 and LSU (partial) rDNA. PCR was carried out in 25 µl volume containing 2.5 µl of 10X buffer, 2.5 µl of 2.5 mM dNTP mix, 0.5 µl of each primer at 10 µM, 0.5 µl of DNA Taq polymerase, 13.5 µl of water without nuclease and 5 µl of DNA template. Amplification was carried out in a thermocycler using a program with the following parameters: 2 min at 95 °C; 37 cycles of 30 s at 95 °C, 30 s at 55 °C, and 60 s at 72 °C; and 5 min at 72 ° C. The PCR products were purified using alkaline phosphatase and exonuclease I and directly cycle-sequenced with ABI BigDye Terminator ver. 3.1 (Applied Biosystems, USA). The sequences obtained were edited using Chromas software ([www.technelysium.com.au](https://www.technelysium.com.au)) and compared to those published in nucleotide databases and the NCBI ([www.ncbi.nlm.nih.gov](https://www.ncbi.nlm.nih.gov)) databases using BLAST [4]. Identification of a species was confirmed if <98% of the sequence identity of the ITS region was shared with the reference sequence from the database. In case of unclear results, morphological characteristics were taken into account.

To prepare fungal food for snails, the 11 most common fungal species (Supplementary Table S1) were selected. New cultures were established, and 0.1 g of mycelium from each 14-day-old culture was collected weekly. Samples were pooled, ground using a mortar, and suspended in 20 ml of water. The mixture was prepared fresh prior to each feeding.

**S2. Detailed rearing conditions**

Throughout all experimental series, individuals were reared singly in ventilated Petri dishes (5 cm diameter) under controlled laboratory conditions (17 °C; 12 h:12 h light:dark photoperiod). To maintain high humidity and prevent desiccation of eggs, snails, and food, Petri dishes were placed inside larger translucent plastic containers (22 × 14 × 12 cm), each holding up to 14 dishes.

Depending on the experimental series, either cotton pads (Experiment 1) or artistic plaster (Experiments 2 and 3) were used as rearing substrates. These substrates were selected to balance moisture retention and minimize accidental ingestion. In all treatments, microbial resources were supplied on small pieces of tissue paper, and eggs or juvenile snails were positioned adjacent to the microbial source to facilitate contact and feeding.

In all treatments, snails were provided with plant litter serving both as substrate and food. Litter was supplemented with a small amount of native soil and supplied in one of three forms:
(1) fresh large pieces of litter,

(2) large pieces of sterilized litter, or

(3) blended, non-sterilized litter.

Litter was collected four days prior to feeding. Large-piece treatments consisted of half a leaf of *Alnus* and two sedge leaves (4 cm each). Sterilized litter was prepared identically but autoclaved for 15 min. Blended litter was produced by homogenizing 300 g of fresh litter in 0.5 L of boiled, cooled water, followed by sieving through a 0.5 mm mesh to obtain a uniform substrate.

**S3. Full experimental volumes and concentrations**

**Experiment 1**

All treatments testing feces-derived bacteria employed cotton pads as the rearing substrate. Fecal bacteria were supplied exclusively in combination with sterilized large-piece litter to isolate their effects from those of naturally occurring litter microbiota. Two bacterial concentrations were tested: OD = 0.01 (SL+FB1) and OD = 0.1 (SL+FB2).

Bacteria were applied onto two tissue-paper discs (0.5 cm diameter) placed approximately 3 cm apart, with 10 µl added per disc (20 µl total per Petri dish). Sterilized litter was placed directly on the cotton pad, and water was added to maintain humidity. Control snails received fresh large-piece litter (LPL) and boiled, cooled water. All treatments were supplemented with dolomite dust as a calcium source. Treatment composition and sample sizes are summarized in Table 1.

**Experiments 2 and 3**

A bacterial suspension at OD = 0.1 was used, based on the higher concentration tested in Experiment 1. When a single bacterial type was applied, a total volume of 20 µl was used; when two bacterial types were combined (e.g. C+LB+FB), each was applied at 10 µl.

In treatments involving fungi or cyanobacteria, a total of 350 µl of microbial material was supplied per Petri dish (Table 1). Bacteria were delivered on tissue-paper discs placed adjacent to sterilized litter, whereas cyanobacteria and fungi were applied directly onto the litter surface using tissue paper as a carrier.

Blended litter was introduced in Experiment 3 to ensure a homogeneous diet and to minimize spatial heterogeneity in food availability while allowing comparison with large-piece litter treatments.

**References**

[1] Johnson LF, Curl EA (1972) *Methods for research on the ecology of soil-borne plant pathogens*. Burgess Publishing, Minneapolis.

[2] Gardes M, Bruns TD (1993) ITS primers with enhanced specificity for basidiomycetes. Molecular Ecology 2:113–118. <https://doi.org/10.1111/j.1365-294X.1993.tb00005.x>

[3] White TJ, Bruns T, Lee S, Taylor J (1990) Amplification and sequencing of fungal rRNA genes. In: Innis MA, Gelfand DH, Sninsky JJ, White TJ (eds) *PCR protocols: a guide to methods and applications*. Academic Press, San Diego, pp 315–322.

[4] Altschul SF, Gish W, Miller W, Myers EW, Lipman DJ (1990) Basic local alignment search tool. Journal of Molecular Biology 215:403–410.

**Supplementary Tables**

Table S1. The most common fungal species selected for the experiment were identified through DNA sequencing and BLAST analysis based on ITS fragments. The species names were verified using the Mycobank database and any name changes are indicated with asterisks. The original names from the NCBI database are as follows: **Acremonium variecolor; **Acremonium persicina.*

| **Isolate** | **Source** | **Identified splecie** | **Blast match sequence** | | |
| --- | --- | --- | --- | --- | --- |
|  |  |  | **Accesion No.** | **Similarity (%)** | **Coverage (%)** |
| SC1_1 | litter | *Paraphaeosphaeria neglecta*  Verkley, Riccioni & Stielow | JX496107 | 100 | 97 |
| SC1_2 | litter | *Cladosporium cladosporioides*  (Fresen.) G.A. de Vries | OP590107 | 100 | 100 |
| SC1_3 | litter | *Epicoccum nigrum*  Link, Mag. Neuesten Entdeck | AJ279448 | 100 | 100 |
| SC2_2 | litter | *Meyerozyma guilliermondii*  (Wick.) Kurtzman & M. Suzuki, | MH545918 | 99.34 | 100 |
| SC2_3 | litter | *Bjerkandera adusta*  (Willd.) Karst. | MF782736 | 99.53 | 100 |
| SC5_2 | litter | *Clonostachys rosea*  (Link) Schroers, Samuels, Seifert & W. Gams | MT732810 | 99.63 | 100 |
| SG1_1 | soil | *Ovicillium variecolor**  (Giraldo, Guarro, Gené & Cano) LW. Hou, L. Cai & Crous | HE608648 | 99.20 | 99 |
| SG1_5 | soil | *Trichoderma harzianum*  Rifai | MZ477246 | 100 | 100 |
| SG1_9 | soil | *Verruciconidia persicina***  (Nicot) L.W. Hou, L. Cai & Crous | OQ513911 | 99.83 | 100 |
| SG2_5 | soil | *Penicillium cosmopolitanum*  Houbraken, Frisvad & Samson | MH859593 | 99.67 | 100 |
| SG2_6 | soil | *Talaromyces penicillioides*  L. Wang | MK837956 | 99.82 | 99 |

**Supplementary Figures**

**Fig. S1.** Kaplan–Meier survival curves for *Vertigo antivertigo* juveniles in Experiment 1, testing the effect of feces-derived bacterial supplementation under sterile litter conditions. Treatments included fresh large-piece litter (Control), sterilized litter (SL), and sterilized litter supplemented with fecal bacteria at OD = 0.01 (SL+FB1) or OD = 0.1 (SL+FB2). Death was defined as the event, whereas individuals reaching the adult stage were censored, as indicated by crosses. The p-value from the log-rank test compares survival distributions across treatments. The risk table indicates the number of individuals remaining under observation in each treatment at successive time points.

**Fig. S2.** Kaplan–Meier survival curves for *Vertigo antivertigo* individuals exposed to cyanobacteria and bacterial resource treatments in Experiment 2. Treatments included fresh large-piece litter (Control), cyanobacteria (C), litter-associated bacteria (LB), cyanobacteria combined with litter-associated bacteria (C+LB), cyanobacteria and litter-associated bacteria with sterilized litter (C+LB+SL), and cyanobacteria and litter-associated bacteria supplemented with feces-derived bacteria (C+LB+FB). Death was treated as the event, and individuals reaching the adult stage were censored, as indicated by crosses. The p-value refers to the log-rank test, and the risk table shows the number of individuals remaining under observation at each time point.

**Fig. S3.** Kaplan–Meier survival curves for *Vertigo antivertigo* individuals exposed to fungal supplementation and substrate-structure treatments in Experiment 3. Treatments included fresh large-piece litter (Control), fungi alone (F), blended litter (BL), and fungi combined with blended litter (F+BL). Death was treated as the event, and individuals reaching the adult stage were censored, as indicated by crosses. The p-value refers to the log-rank test, and the risk table shows the number of individuals remaining under observation at each time point.
